# Breaking the Restriction Barriers and Applying CRISPRi as a Gene Silencing Tool in *Pseudoclostridium thermosuccinogenes*

**DOI:** 10.3390/microorganisms10040698

**Published:** 2022-03-24

**Authors:** Joyshree Ganguly, Maria Martin-Pascual, Diego Montiel González, Alkan Bulut, Bram Vermeulen, Ivo Tjalma, Athina Vidaki, Richard van Kranenburg

**Affiliations:** 1 Corbion, 4206 AC Gorinchem, The Netherlands; jganguly@its.jnj.com; 2 Laboratory of Microbiology, Wageningen University and Research, 6708 WE Wageningen, The Netherlands; maria.martinpascual@wur.nl (M.M.-P.); bm.vermeulen@gmail.com (B.V.); ivo.tjalma@wur.nl (I.T.); 3 Department of Genetic Identification, Erasmus MC University Medical Center Rotterdam, 3015 GD Rotterdam, The Netherlands; d.montielgonzalez@erasmusmc.nl (D.M.G.); a.vidaki@erasmusmc.nl (A.V.); 4 Fontys University of Applied Sciences, 5612 AR Eindhoven, The Netherlands; alkan1998@hotmail.com

**Keywords:** *Pseudoclostridium thermosuccinogenes*, genetic accessibility, in vivo methylation, CRISPRi, malic enzyme

## Abstract

*Pseudoclostridium thermosuccinogenes* is a thermophilic bacterium capable of producing succinate from lignocellulosic-derived sugars and has the potential to be exploited as a platform organism. However, exploitation of *P. thermosuccinogenes* has been limited partly due to the genetic inaccessibility and lack of genome engineering tools. In this study, we established the genetic accessibility for *P. thermosuccinogenes* DSM 5809. By overcoming restriction barriers, transformation efficiencies of 10^2^ CFU/µg plasmid DNA were achieved. To this end, the plasmid DNA was methylated in vivo when transformed into an engineered *E. coli* HST04 strain expressing three native methylation systems of the thermophile. This protocol was used to introduce a ThermodCas9-based CRISPRi tool targeting the gene encoding malic enzyme in *P. thermosuccinogenes*, demonstrating the principle of gene silencing. This resulted in 75% downregulation of its expression and had an impact on the strain’s fermentation profile. Although the details of the functioning of the restriction modification systems require further study, in vivo methylation can already be applied to improve transformation efficiency of *P. thermosuccinogenes.* Making use of the ThermodCas9-based CRISPRi, this is the first example demonstrating that genetic engineering in *P. thermosuccinogenes* is feasible and establishing the way for metabolic engineering of this bacterium.

## 1. Introduction

Metabolic engineering and synthetic biology are currently driving the development of genetically improved microorganisms to produce compounds of interest. To this end, two main different approaches have been applied. On one hand, mesophilic model organisms, such as *Escherichia coli* or *Saccharomyces cerevisiae* have been engineered to produce non-native compounds by introducing heterologous pathways in an efficient, rapid, and high-throughput manner. However, challenges such as cofactor imbalance, genetic instability, or toxicity due to heterologous gene expression need to be tackled for optimal performance [1,2]. Alternatively, non-model production strains with their native ability to produce a target compound or to endure harsh conditions have been exploited. In this case, the genetic accessibility and the availability of an extensive genetic toolbox are crucial factors when genetic engineering is needed to further improve production titers and yields.

Over the last decades, non-model thermophilic microorganisms such as *Clostridium* species have been exploited as whole-cell biocatalysts [3]. Thermophiles are beneficial over mesophiles with better substrate and product solubility, increased reaction rates together with reduced cooling costs, and facilitated recovery of volatile products [4]. In addition, thermophilic anaerobes such as clostridia are preferred over aerobes that suffer the decreasing productivities at low oxygen transfer rates and require costly aeration systems in industrial fermentations [5]. Despite the abovementioned advantages of using thermophilic clostridia as microbial cell factories, limitations such as genetic inaccessibility and limited genome editing tools hinder research on clostridial physiology and metabolism [3,6]. The reasons for genetic inaccessibility are the inability of the plasmid DNA to penetrate the bacterial cell envelope—notably a thick layer of peptidoglycan is characteristic to Gram-positive bacteria—and/or its intracellular degradation due to the presence of phage defense systems, such as restriction-modification (RM) systems [1,7,8]; CRISPR-Cas systems (clustered regularly interspaced short palindromic repeats loci, coupled to CRISPR-associated genes) [7,8,9]; and the defense island system associated with restriction-modification (DISARM) system [10].

The anti-phage mechanisms avoid not only phage invasion or gene transfer among bacteria, but also genetic manipulation as the active RM systems cleave foreign DNA that is not correctly methylated. Most of the RM systems comprise two types of enzymes, a DNA methyltransferase (MTase) and a restriction endonuclease (REase). The MTase methylates specific nucleotides in a DNA sequence within the host’s genome, discriminating non-self and self-DNA and protecting the latter from digestion. The REase recognizes the same sequence as the MTase and cleaves the foreign DNA that lacks proper methylation. Four different RM systems, type I, II, III, and IV, have been described, differing in enzyme composition and mode of action. Type I is a multi-subunit complex consisting of two-restriction (R), two-modification (M), and a specificity (S) subunit (R_2_M_2_S). It requires all subunits (R_2_M_2_S) for restriction activity. In contrast, the Type II RM system involves only two enzymes: REase and the MTase. Type III system comprises two R and M subunits. Contrary to all other RM systems, type IV lacks the MTase enzyme. The REase recognizes non-specific and variable target sites, which are methylated, hydroxy-methylated, or glucosyl-hydroxy methylated [7,11,12]. As a result, RM systems are responsible for an all-or-nothing effect in the transformation efficiency of some difficult to transform-bacteria such as several *Clostridium* or *Bacillus* species. Subsequently, they have greatly hindered the study of the clostridial physiology and its exploitation for industrial applications [13].

**Figure 1:**
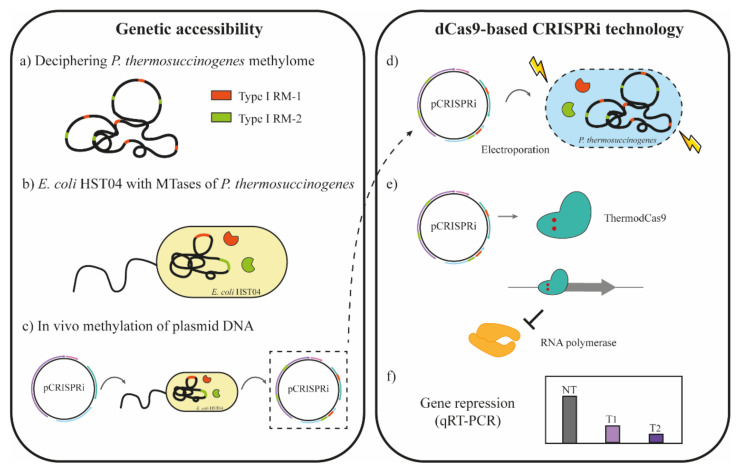
Schematic overview for in vivo methylation and CRISPRi application for *P. thermosuccinogenes* DSM 5809. (**a**) The genome sequence and methylome of *P. thermosuccinogenes* was used to identify the native restriction modification (RM) systems. Three different RM systems were identified in *P. thermosuccinogenes* DSM 5809, but only two are depicted in the figure. (**b**) A set of *E. coli* HST04 derivative strains was constructed by cloning of the methyltransferase genes (orange and green) of *P. thermosuccinogenes*. (**c**) The ThermodCas9 plasmid of interest (pCRISPRi) was introduced in these *E. coli* strains by electroporation. The methylated pCRISPRi plasmid DNA was isolated from this strain and (**d**) electroporated into *P. thermosuccinogenes*. In this way, the pCRISPRi presents a methylation pattern similar to that of the genome of *P. thermosuccinogenes*, overcoming the restriction barrier. (**e**) pCRISPRi expresses the ThermodCas9 and the sgRNA, downregulating the gene of interest upon recognizing and binding the target DNA (promoter region). Transcription is blocked since the Cas9 protein physically interferes with the RNA polymerase. (**f**) Gene repression of desired gene was evaluated by qRT-PCR.

Despite the difficulty to genetically transform clostridia, one strategy that has been successfully used is mimicking host methylation patterns to overcome the restriction barrier in the bacterium that harbors multiple RM systems [14,15]. Such strategy requires an *E. coli* strain deficient in characterized RM systems and orphan MTases (*dam^−^*, *dcm^−^*, *hsdRMS^−^*), such as *E. coli* HST04, because the target bacteria might express not only type I, II, and III RM systems but also type IV systems [11,15]. Thus, the plasmid of interest is not methylated and therefore can be propagated. Otherwise, it presents methylation patterns different to the native DNA, being consequently recognized as foreign DNA and cleaved. To produce host-mimicking DNA, prior identification of native RM systems and their recognition sequences was crucial. Once the recognition sequences on transforming plasmids were identified, they were protected via methylation. This methodology to overcome the restriction barrier for genetic accessibility of non-model organisms is described in detail in Figure 1.

There are two main approaches to methylate DNA, in vitro and in vivo, depending on the type of RM system [11]. In vitro methylation uses commercially available methyl transferases prevalently used for type II RM systems. Although it is a simple and highly efficient strategy, the main drawback is the limited number of methyl transferases. Alternatively, cell extracts can be used for in vitro methylation. However, such strategy may suffer from lack of co-factors and does not work equally efficient for all the systems. In contrast, in vivo methylation as shown in Figure 1 consists of expressing the genes encoding MTases in the genome of an *E. coli* lacking all known RM systems and orphan MTases. While type I methylation can be reproduced by simultaneous expression of M and S subunit genes, type II and III methylation can be mimicked by expressing M subunit genes [12,16,17]. In addition, bioinformatics analysis using single-molecule real-time (SMRT) sequencing from PacBio [12,18] or Nanopore Minion sequencing [19] can be used to help understand the methylome of the strains. This combination of methods assisted in overcoming restriction modification systems to enhance genetic accessibility in various bacteria [19,20,21].

*Pseudoclostridium thermosuccinogenes* is a thermophilic anaerobe that produces succinate and acetate, as major products, and formate, lactate, and ethanol as minor products, from sugars such as inulin, glucose or xylose [16,17,22]. Accordingly, to become attractive as a succinate producer, *P. thermosuccinogenes* needs to be optimized by genome engineering and requires genetic accessibility. Genetic engineering of *P. thermosuccinogenes* strains has not been described to date. *Hungateiclostridium thermocellum* is a close relative for which genetic tools have been developed. Repurposing the CRISPR-Cas9 technology has established the method for efficient genome editing tools in various organisms [20,21,23,24,25]. Recently, two thermophilic Cas9 variants GeoCas9 [26] and ThermoCas9 [27] were applied in *H. thermocellum*. The latter was applied as ThermodCas9 for CRISPR interference (CRISPRi) to downregulate phosphotransacetylase and lactate dehydrogenase genes [28]. Another recent study developed the Type II (GeoCas9) CRISPR-Cas system together with recombineering machinery for efficient genome editing purposes in the same bacteria [29].

In general, in CRISPR-Cas systems the active Cas9 protein-sgRNA ribonucleoprotein complex cleaves the DNA. In contrast, CRISPRi systems cannot cleave the DNA as a result of two point mutations present in the Cas9 protein. The catalytically inactive Cas protein binds the DNA complementary to the sgRNA, blocking the RNA polymerase and repressing the transcription of the target gene. CRISPRi is particularly suitable to target metabolic genes or pathways which require a basal expression. Additionally, it serves as a powerful method for fast assessment of the possible impact of the intended genetic modifications in the microbial metabolism [29,30,31,32,33,34].

In this study, we developed a protocol for transformation of *P. thermosuccinogenes* DSM 5809 by electroporation. We applied an in vivo methylation pipeline mimicking DNA methylation patterns of *P. thermosuccinogenes* DSM 5809 in an engineered *E. coli* HST04 strain expressing the three native type I methylation systems and lacking any other known RM systems to avoid unintended modifications. Plasmids prepared from this host showed increased transformation efficiency, apparently, escaping the native restriction systems. After successfully overcoming the restriction barriers of this strain, as a proof of concept, we developed a CRISPRi silencing tool for *P. thermosuccinogenes* DSM 5809 (Figure 1). To conclude, this research demonstrates for the first time the genetic accessibility of *P. thermosuccinogenes* followed with the development of a CRISPRi tool for downregulating the malic enzyme, unraveling a way for prevalent metabolic engineering of this thermophile.

## 2. Materials and Methods

### 2.1. Bacterial Strains and Growth Conditions

All the strains with their respective characteristics used in the present study are described in Table S1, Supplementary Materials. Chemically competent *E. coli* DH5α was used for cloning purposes. *E. coli* JM110 [35], Acella (Chemically Competent cells, Edge Bio), and HST04 (Stellar^TM^ Competent Cells, *dam^−^*/*dcm^−^*, Takara Bio) were used for plasmid propagation and isolation before *P. thermosuccinogenes* DSM 5809 transformation. *E. coli* strains were cultured in Lysogeny Broth (LB) medium at 37 °C and 200 rpm, unless otherwise specified. Antibiotics were added when required, at the following concentrations: chloramphenicol, 20 μg/mL; ampicillin, 150 μg/mL; kanamycin, 20–50 μg/mL. *P. thermosuccinogenes* DSM 5809 was obtained from the German Collection of Microorganisms and Cell Cultures (DSMZ). Media preparations used for P. thermosuccinogenes are described in Section S1, Supplementary Materials. *P. thermosuccinogenes* DSM 5809 wild type and transformants were grown anaerobically in CP medium adapted from [36]. The final volume of 50 mL medium was dispensed in serum bottles under 80:20 N_2_/CO_2_ atmosphere with ∼70 kPa overpressure and then autoclaved. The wild type and transformants were obtained in CTFUD agar plates adapted from Olson et al. and grown at 50–60 °C range [37]. Glucose as a carbon source was also autoclaved separately and added later to a final concentration of 5.0 g/L. If needed thiamphenicol, 6 μg/mL was added. For *P. thermosuccinogenes* spore purification, it was grown anaerobically at 60 °C overnight in CP medium. In order to trigger sporulation, 1 mL of pre-culture was added to sporulation medium adapted from Yang et al. and Mearls et al. [38,39], cultured at 55 °C for 2 days.

### 2.2. Preparation and Purification of P. thermosuccinogenes DSM 5809 Spores

*P. thermosuccinogenes* DSM 5809 was grown anaerobically at 60 °C overnight in CP medium. For sporulation, the Modified AEA Sporulation Medium Base (catalogue number 17170, Sigma Aldrich) was prepared, and 30 mL dispensed into anaerobic bottles under 80:20 N_2_/CO_2_ with ∼70 kPa overpressure and then autoclaved. After autoclavation, the medium was supplemented with 0.4% glucose solution, freshly prepared and filter sterilized, 0.19% of Na_2_CO_3_, and 2.29% CoCl_2_·6H_2_O. Then, 0.88% of freshly prepared sodium ascorbate solution was added. One milliliter of the pre-culture was added to the sporulation medium and incubated at 55 °C. Once spores were formed, 1 mL of pre-culture from sporulation medium was inoculated into 50 mL CP medium and grown for 2 days at 60 °C. When mature spores were highly predominant, spore suspension was harvested by centrifugation at 4800*× g* for 15 min. Cell pellets were resuspended and gently layered on top of 5 mL of the 50% Histodenz solution [38,39]. The Histodenz gradient together with spore suspension was centrifuged at 3000*× g*, 20 °C for 60 min. Spore pellets were collected, resuspended in 15 mL Milli-Q water, centrifuged at 1500*× g*, 20 °C for 30 min and supernatants were discarded. Mature spores pellets were resuspended in 10 mL Milli-Q water. Samples were examined by phase contrast microscopy (Figure S1, Supplementary Materials). One milliliter aliquots of mature spores were stored at −80 °C.

### 2.3. Plasmid Construction

All PCR reactions for cloning were performed with the NEB Q5^®^ High-Fidelity DNA polymerase according to the manufacturer’s instructions (M0491). PCR products were subjected to 1% w/v agarose gel electrophoresis and isolated using Zymoclean^TM^ Gel DNA Recovery kit. Plasmids were built using the NEBuilder^®^ HiFi DNA assembly cloning kit according to the manufacturer’s protocol. The plasmids developed in this study, and the primers used to construct them, together with a detailed overview of the setup, are presented in Table S2, Supplementary Materials. For genetic accessibility experiments, *E. coli*–*Bacillus* shuttle vector pNW33N was used, containing an origin of replication that functions in *H. thermocellum* and a chloramphenicol/thiamphenicol antibiotic resistance marker. For the CRISPRi experiments, the pThermoCas9i plasmid with rRNA promoter for sgRNA expression, developed in a previous work [28] was used as a template for the construction of a plasmid targeting the malic enzyme (CDQ83_17115_S2: 987621_988793) in *P. thermosuccinogenes* DSM 5809. The sequences of the non-targeting and targeting spacers are listed in Table S3, Supplementary Materials.

*P. thermosuccinogenes* DSM 5809 genome encodes multiple RM systems. Three type I and one type III RM systems have been identified (Table 1). To increase the strain’s genetic transformability, pThermoCas9i plasmids were analyzed for the presence of their recognition sites. The presence of the recognition sites was abolished by introducing point mutations. Five nucleotides were replaced by the most suitable synonymous substitutions (Table S4, Supplementary Materials), taking into consideration the codon usage bias of *P. thermosuccinogenes* DSM 5809 (Table S5, Supplementary Materials).

All plasmids were introduced by heat-shock in chemically competent *E. coli* DH5α cells. Single colonies present in the selective medium were examined. They were grown in LB cultures with the appropriate antibiotic. Plasmids were isolated by using the GeneJET Plasmid Miniprep Kit (Thermo Scientific) and confirmed by standard sequencing from Macrogen (MACROGEN Inc. DNA Sequencing Service; Amsterdam, The Netherlands) using the primers shown in Table S6, Supplementary Materials. Plasmids were electroporated in *E. coli* HST04 and *E. coli* JM110 and reisolated before being electroporated in *P. thermosuccinogenes*. Because of the exceptionally high concentrations of plasmid DNA required for the transformations in clostridial species, specifically in *P. thermosuccinogenes*, midiprep was performed to efficiently extract the plasmid from the intermediate *E. coli* strains, according to the manufacturer’s instructions of ZymoPURE II™ plasmid isolation kit.

### 2.4. Electroporation of P. thermosuccinogenes DSM 5809

Prior to genetic transformation by electroporation, 1 mL of dormant mature spores, stored at −80 °C, was heat activated at 80 °C for 15 min. Heat activation decreases the lag phase, allowing more spores to germinate [40]. The activated spores were in turn inoculated into 50 mL CP medium. Spores germinated overnight at 60 °C. Next day, 4–6 mL of the overnight culture was reinoculated into fresh CP medium, with OD_600_ ~ 0.1. The bacterial culture was grown anaerobically at 60 °C to mid-log phase until OD_600_ ~ 0.6 and the culture was kept at room temperature for 20 min. To harvest the cells, the culture was brought into the anaerobic chamber and transferred anaerobically to a 50 mL Greiner tube. Closed tubes were taken from the anaerobic chamber and cells were harvested by centrifugation at room temperature and 4800*× g* for 20 min. Twenty milliliters of the 10% (*v*/*v*) glycerol wash buffer was added without disturbing the pellet and centrifuged at room temperature, 4800*× g* for 15 min. The supernatant was removed, and the washing step with glycerol wash buffer was repeated. Finally, cells were resuspended in 100–500 μL of 10% (*v*/*v*) glycerol. Eighty-microliter aliquots of cells were distributed in 1.5 mL Eppendorf tubes; 1–4 μL of plasmid DNA, containing 4 μg, was added to each aliquot. The plasmids were previously isolated from *E. coli* HST04 (*dam^−^*/*dcm^−^*) to ensure the transfer of unmethylated plasmids. The samples were transferred to the anaerobic chamber, and the mixture was pipetted into a 2-mm gap electroporation cuvette. A single exponential decay pulse was applied using a Gene Pulser X-Cell (Bio-Rad) set at 1.8 kV, 200 Ω, and 25 μF. Immediately after the electrical pulse, cells were resuspended in 4 mL CP media, and incubated anaerobically at 50 °C inside a dry incubator for 10–12 h; 100 μL, 2 mL, and remaining of the transformed cells were plated in CTFUD media containing 6 μg/mL of thiamphenicol and 5 g/L of glucose. The agar plates were placed inside a 2.5 L anaerobic jar (ThermoFisher) with the Oxoid^TM^ AnaeroGen^TM^ 3.5 L anaerobic bag (ThermoFisher). The anaerobic jar, containing the transformed plates was placed inside a dry incubator at 50 °C. If the transformation succeeded, after 3–5 days colonies were visible. For the transformation of CRISPRi plasmids, the protocol was modified with the electrical settings for electroporation. A single square wave pulse was applied at 1.0 kV and 5 milliseconds.

### 2.5. Prediction of P. thermosuccinogenes DSM 5809 Methylome Based on PacBio Sequence Information

Detection of bacterial base modifications was performed in the PacBio SMRT portal (v2.3) using the ’RS_Modification_and_Motif_Analysis.1‘ workflow on the PacBio dataset from the DSM 5809 genome assembly PRJNA388583 [21]. This workflow consists of (i) filtering the data, (ii) mapping the filtered sequences against a reference genome, (iii) consensus calling of the mapped reads, and (iv) detecting the base modifications (indels, single nucleotide variants, and multiple nucleotide variants) and motifs. For the identification of 6-methyladenine DNA base modifications, 25× sequencing coverage per strand was done.

### 2.6. Generating E. coli HST04 Strains with Methyltransferases Genes of P. thermosuccinogenes

**Figure 2:**
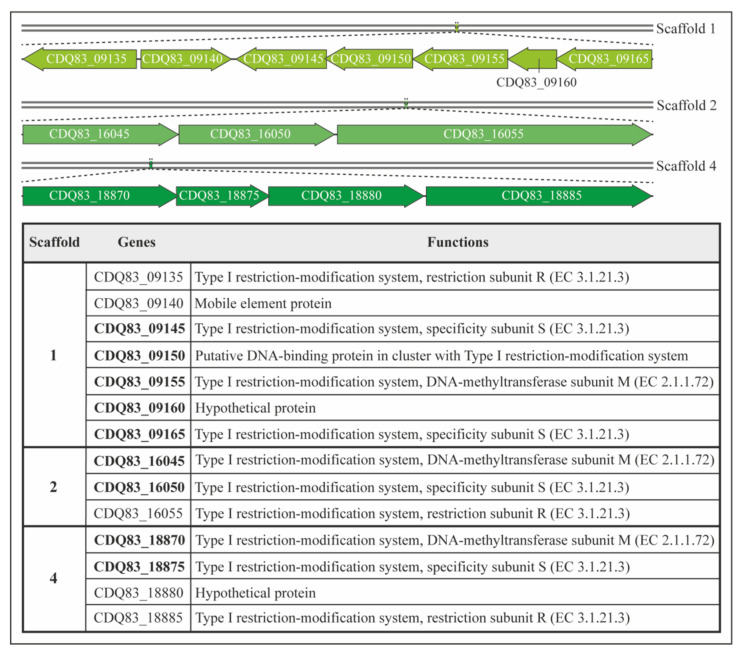
Type I RM systems in the genome of *P. thermosuccinogenes* DSM 5809. Genetic maps with gene functions of the different type I RM systems in *P. thermosuccinogenes*. Scaffold 1 harbors the gene cluster CDQ83_09135, CDQ83_09140, CDQ83_09145, CDQ83_09150, CDQ83_09155, CDQ83_09160, and CDQ83_09165. Scaffold 2 harbors the gene cluster CDQ83_16045, CDQ83_16050, and CDQ83_16055. Scaffold 3 harbors the gene cluster CDQ83_18870, CDQ83_18875, CDQ83_18880, and CDQ83_18885. The ORFs from each Type I RM systems cloned in *E. coli* are highlighted in bold.

Genes encoding three different putative type I RM systems in *P. thermosuccinogenes* DSM 5809 were introduced in the genome of *E. coli* HST04 using the lambda Red system enabled by the pKD46 plasmid [41]. Three functional cassettes were constructed. The primers used to construct them, together with a detailed overview of the setup, are presented in Table S7, Supplementary Materials. Cassette S1 comprised the genes of an operon, encoding one M and two S subunits from a type I RM system and an additional unknown protein, annotated in scaffold 1 (CDQ83_09145-CDQ83_09165). Cassette S2 contains the genes encoding the M and S subunits from a type I RM system, annotated in scaffold 2 (CDQ83_16045 and CDQ83_16050). Cassette S4 harbors the genes encoding the M and S subunits from the type I RM system, annotated in scaffold 4 (CDQ83_18870-CDQ83_18875). The R subunits were not included in the cassettes. All the genes present in the cassettes are indicated in bold in Figure 2.

The functional cassettes were flanked by 100 bp long homology arms for the disruption of the targeted locus in the *E. coli* chromosome. The homology arms correspond to the upstream (5′ end) and downstream (3′ end) sequence, separated from each other ~ 1 kb in the insertion site.

Consequently, the bifunctional isocitrate dehydrogenase kinase/phosphatase gene (08395), the lacZ gene, and the transcriptional regulator gene *arsR* (11130), and the arsenic transporter gene (11125) were disrupted (Table S1).

The synthetic linear cassettes are composed of two elements: DNA methyltransferase expression module and *FRT*-flanked antibiotic resistance unit. The DNA methyltransferase expression module carries an inducible promoter, which controls the expression of the methylation genes and the T7 terminator, allowing efficient transcription termination. Aside from the methylation module, the antibiotic resistance unit is composed of an antibiotic resistance gene which is flanked by the *FRT* sites (Figure S2, Supplementary Materials). Therefore, the *FRT*-flanked antibiotic selection unit could be excised by transformation of the flippase (FLP) expression vector, pCP20 into the *E. coli* HST04, harboring the cassette. These engineered *E. coli* HST04 hosts were further used for transformations in *P. thermosuccinogenes*. For induction of different RM systems, individual *E. coli* HST04 strains harboring type I RM system_S1 were cultured in LB media with 0.1 mM IPTG, *E. coli* HST04 strains harboring type I RM system_S2 were cultured in LB media with 0.1% arabinose, and *E. coli* HST04 strains harboring type I RM system_S4 were cultured in LB media with 1 mM m-tuloic acid at 37 ºC and 200 rpm overnight. *E. coli* HST04 strains harboring the three cassettes were cultured in LB medium with 0.1 mM IPTG, 0.1% arabinose and 1 mM m-tuloic acid at 37 °C and 200 rpm overnight.

### 2.7. RNA Isolation and First Strand cDNA Synthesis

RNA isolation of *P. thermosuccinogenes* transformants, harboring the pThermoCas9i plasmids was performed using 5 mL of overnight cultures at an OD_600_ ~ 1.0. The RNA isolation for qRT-PCR was adapted from Ganguly et al. [28]. The Maxwell 16 LEV Total RNA Cells Kit was used to obtain RNA from the transformants. The quality and the concentration of the purified RNA were determined using a NanoDrop spectrophotometer and the material was stored at −20 °C. The first strand cDNA synthesis was performed with SuperScript^TM^ III Reverse Transcriptase (Invitrogen) following the manufacturer’s instruction. The primers BG11642 and BG11643 were used to amplify 169 bp of the sgRNA and the primers BG11636 and BG11637 were used to amplify 282 bp of the ThermodCas9 using the NEB Q5^®^ High-Fidelity DNA polymerase.

### 2.8. Quantitative Real Time PCR

The qRT-PCR was performed by using the iQ^TM^ SYBR^®^ Green Supermix from Bio-Rad. The cDNA samples were diluted in sterile Milli-Q water. The amount of cDNA used as a template was equivalent to 50 ng of RNA. The housekeeping gene used to measure the relative expression was 16S rRNA of *P. thermosuccinogenes* DSM 5809. The primers used to amplify 16S rRNA and malic enzyme gene of *P. thermosuccinogenes* were BG10427, BG10428 and BG18976, BG18977, respectively (Table S6, Supplementary Materials). The qRT-PCR was run in a Bio-Rad C1000 Thermal Cycler.

### 2.9. High-Performance Liquid Chromatography

A high-performance liquid chromatography (HPLC) system ICS-5000 was used for the organic acids and ethanol quantification. The system had an Aminex HPX 87H column from Bio-Rad Laboratories and was equipped with a UV1000 detector operating at 210 nm and an RI-150 40 °C refractive index detector. The mobile phase consisted of 0.16 N H_2_SO_4_ and the column was operated at 0.8 mL/min. All samples were diluted 4:1 with 10 mM DMSO in 0.01 N H_2_SO_4_. *P. thermosuccinogenes* non-targeting control and malic enzyme gene silencing transformants were grown in CP medium (5 g/L yeast extract) for 2 days and samples were taken at different time points from OD_600_ > 1.0 for analysis with HPLC. Sugars (cellobiose, glucose, ethanol, and glycerol) and organic acids (malic acid, pyruvic acid, acetic acid, lactic acid, succinic acid, and formic acid) were used as standards with a concentration range between 1.25 and 25 mM.

## 3. Results

### 3.1. Initial Transformation Steps for P. thermosuccinogenes DSM 5809

An initial transformation protocol was developed using plasmid pNW33N, which was known to replicate in *H. thermocellum* DSM 1313 [37]. Initially, we examined the effect of *dam* and *dcm* methylation on electroporation. Plasmids were extracted from *E. coli* strains with different methylomes DH5α (*dam*^+^/*dcm*^+^), Acella (*dam*^+^/*dcm*^−^), JM110 (*dam*^−^/*dcm*^−^), and HST04 (*dam*^−^/*dcm*^−^/*Δ*(*mrr-hsdRMS-mcrBC*)) to prepare methylated/unmethylated plasmid DNA (Table S8, Supplementary Materials). Several rounds of electro-transformation experiments were performed using plasmid pNW33N isolated from *E. coli* DH5α, Acella, JM110, and HST04 strains with conditions adapted from Olson et al. for *H. thermocellum* [37] (for details, see Materials and Methods). For our transformation protocol, the major modifications were: (i) the preparation of electrocompetent cells, carried out at room temperature; (ii) the electroporation settings, adjusted to 1.8 kV, 200 Ω, and 25 μF, exponential decay pulse; and (iii) the recovery time of cells in CFTUD liquid medium without any antibiotics for 10–12 h after which the cells were plated on CFTUD-Tm agar medium. The initial results showed a few thiamphenicol (Tm^R^) resistant colonies only for plasmids isolated from non-methylating *E. coli* JM110 and HST04 strains. The Tm^R^ colonies were verified by colony PCR and plasmid DNA was isolated from *P. thermosuccinogenes* and retransformed in *E. coli* DH5α (Figure S3, Supplementary Materials) confirming the presence of pNW33N in *P. thermosuccinogenes* transformants. This shows the importance of using unmethylated DNA for successful transformation in *P. thermosuccinogenes*. Secondly, we observed poor reproducibility and efficiency between independent transformation experiments. Thus, we aimed to improve both the abovementioned factors by various strategies to overcome restriction barriers as described in next sections.

### 3.2. Bioinformatics Analysis of Restriction Modification Systems in P. thermosuccinogenes

*P. thermosuccinogenes* DSM 5809 genome sequence was assembled using data from Illumina HiSeq and PacBio sequencing [22]. The annotation included genes annotated as putative restriction systems and DNA methyltransferases. In the annotated sequence data, three type I restriction systems and one type III system were identified that could negatively impact the transformation efficiency. SMRT sequencing data were further analyzed, as described in Methods, to identify all base modifications and to find modified sequence motifs. Originally, five different motifs were identified. However, two motifs, ‘GATNNNNCTC’ and ‘DGAGNNNNATC’, were complementary to each other except for the addition of a D (A, G, or T) to one motif. Therefore, we hypothesized that these two motifs were both halves of a single motif and part of the same type I RM system (Table 1). Based on these data, we concluded that *P. thermosuccinogenes* DSM 5809 has three type I RM systems. In addition, the methylome analysis revealed the presence of a type III RM recognition motif (‘CGAG’). The genome sequence contains three gene clusters of the predicted type I RM systems in different scaffolds. Scaffold 1 comprises genes for two S subunits, one R and one M subunit, and three additional elements (a mobile element protein, a putative DNA binding protein, and a hypothetical protein). Scaffold 2 and Scaffold 4 each have genes for a single S, R, and M subunit, respectively (Figure 2).

### 3.3. Strategies to Overcome Restriction Barrier to Improve Transformation Efficiency

**Figure 3:**
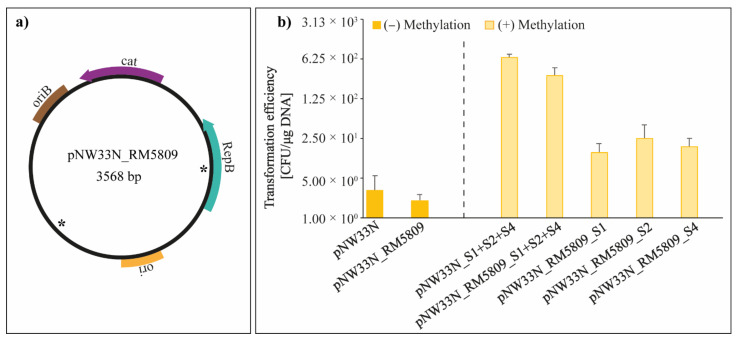
Strategies to enhance transformation efficiency by breaking the restriction barriers. (**a**) Schematic illustration of plasmid pNW33N_RM5809 with mutated RM recognition sites marked (*); ori–pUC19 origin of replication for plasmid propagation in *E. coli;* oriB–pNW33N origin of replication for plasmid propagation in *P. thermosuccinogenes*; *repB*–replication protein for pNW33N origin of replication; *cat*–chloramphenicol acetyl-transferase, provides resistance to chloramphenicol and thiamphenicol. (**b**) Transformation efficiency of *P. thermosuccinogenes* using the plasmids pNW33N and pNW33N_RM5809 isolated from modified *E. coli* HST04 strains consisting of all methylation patterns. Values represent the mean and the standard deviation of three independent plasmid DNA isolations that were used for each electroporation.

The pNW33N plasmid was examined for the presence of putative RM recognition sites retrieved from the methylome analysis of *P. thermosuccinogenes* DSM 5809 (Table S4, Supplementary Materials). We identified three recognition sites of motif 2 (Table 1) on the plasmid that might potentially hinder the transformation of the plasmid. Since few transformants were obtained previously, the hypothesis was that some transformed plasmids can escape restriction and become methylated by the native methyltransferases, enabling propagation. This might also explain the low reproducibility and efficiency of the transformation experiments. To test this hypothesis, two strategies were followed. Firstly, the detected recognition sites on pNW33N were mutated, so they can no longer be recognized by the corresponding RM systems. Secondly, the recognition sequences were protected by in vivo methylation. Thus, *E. coli* strains were engineered by introducing native genes encoding type I RM systems from *P. thermosuccinogenes* DSM 5809. For the first approach, the three different recognition sites that were detected are in the *repB* gene, the promoter region of the chloramphenicol acetyltransferase (*cat*) gene, and the backbone vector (Table S4, Supplementary Materials). The *P. thermosuccinogenes* codon bias was established using the Codon Usage program of the Sequence Manipulation Suite (SMS) [42] and considered to apply synonymous mutations with the codon usage closest to the original codon usage (Table S5, Supplementary Materials). The site that was present in the promoter region of the *cat* gene was not modified to avoid any potential side effect to the proper transcription of the chloramphenicol resistance gene. Thus, the plasmid pNW33N_RM5809 (pNW33N lacking two RM recognition sites, except the one present in the promoter of the *cat* gene, Figure 3b) was electroporated into *P. thermosuccinogenes* to evaluate the transformation efficiency. The plasmids pNW33N_RM5809 and pNW33N (control), isolated from *E. coli* JM110 or HST04, did not show any significant difference in transformation efficiency, but the reproducibility was improved for pNW33N_RM5809 (Table S8, Supplementary Materials). Reproducibility in this context means that after introduction of the mutations to the plasmid, three independent plasmid DNA isolations were used for each electroporation. This resulted in the same order of magnitude of transformation efficiencies, indicating the results and experiments were reproducible. In contrast, the transformation results were not always reproducible using plasmid pNW33N and frequently did not yield any transformants.

As a second strategy, we attempted to mimic the host’s methylation patterns in *E. coli* by in vivo methylation of the RM recognition sequences of the plasmid DNA. This strategy has been shown to significantly increase the transformation efficiency in various bacteria [12,17,22,43] for which the highest improvement of transformation efficiency was a 10^4^-fold increase (to 3 × 10^6^ CFU/µg plasmid DNA) reported for *B. amyloliquefaciens* [15].

Linear cassettes, carrying different predicted type I methylation systems identified in the genome of *P. thermosuccinogenes* DSM 5809, were designed by making use of three different inducible promoters that would control the expression of the methylation genes. To know which methylation system had the strongest effect in the transformation efficiency, we engineered three different strains with individual RM systems or a single strain with the three RM systems together. Three cassettes (referred as S1, S2, and S4, Figure S2 and Table S7) composed of the different type I RM systems from the three scaffolds of *P. thermosuccinogenes* were successfully generated via overlapping PCR and integrated in the genome of *E. coli* HST04 by Lambda Red recombineering. The generated *E. coli* HST04 strains with the integrated cassettes (S1, S2, S4, or S1-S2-S4) were used to methylate the pNW33N and/or pNW33N_RM5809 (Figure 3b) plasmids to mimic the native methylation patterns of *P. thermosuccinogenes* DSM 5809 prior to electroporation (Figure 3a). In addition, we tested if easy access MinION sequencing data could be used to identify type I methylation patterns of DNA isolated from the different *E. coli* HST04 hosts and correlate these with the integrated cassettes.

The preliminary data from Minion sequencing and methylation analysis indicated that the *E. coli* HST04-S1 strain has significant ^6m^A methylation in the motif ‘GATNNNNCTC’ (Bonferroni < 1.05 × 10^−19^) compared to the wild type *E. coli* HST04 strain and other engineered strains (Table S9 and Section S2. Materials and Methods, Supplementary Materials). This may explain the improved transformation efficiency of pNW33N_RM5809 that still has one ‘GATNNNNCTC’ recognition site left. For reasons yet unknown, the motif was not retrieved from the HST04-S1-S2-S4 plasmid DNA transformants, nor was any other motif found to be enriched in any of the four strains. The effect on transformation efficiency of using the different intermediate *E. coli* HST04 strains to methylate plasmid DNA showed that the combination of the three methylation systems, S1, S2, and S4, on pNW33N and pNW33N_RM5809 has the highest effect on the transformation efficiency with an increase of two orders of magnitude, corresponding to 6.6 × 10^2^ CFU/µg DNA and 3.2 × 10^2^ CFU/µg DNA, respectively (Figure 3b). Unexpectedly, plasmid DNA isolated from *E. coli* strains expressing any of each single methylation system all had a similar increase in transformation efficiency of one order of magnitude (Figure 3b), while we expected that plasmid DNA isolated from *E. coli* HST04-S1 would result in transformation efficiencies in the range of plasmid DNA isolated from *E. coli* HST04-S1-S2-S4. In addition, we anticipated plasmid DNA isolated from *E. coli* HST04-S2 or HST04-S4 would have transformation efficiencies comparable to non-in-vivo-methylated plasmid DNA. These results indicate an underlying unknown molecular mechanism of these type I RM systems that provides an additive effect on increasing the transformation efficiency.

### 3.4. CRISPRi as a Silencing Tool for P. thermosuccinogenes

**Figure 4:**
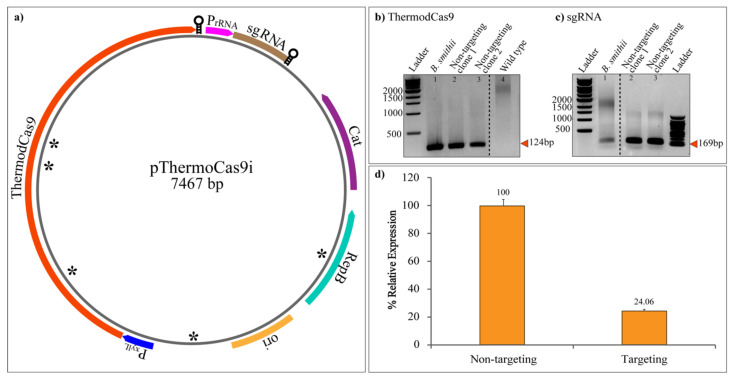
Transcriptional repression of malic enzyme gene by CRISPRi of *P. thermosuccinogenes* transformants. (**a**) Schematic representation of plasmid pThermoCas9i (Mougiakos et al. 2017b). The *thermodcas9* gene under control of the *B. smithii xylL* promoter; sgRNA-expressing module under control of the native 16S/23S intergenic rRNA promoter from *H. thermocellum*; pNW33N backbone; asterisks (*) represent the regions with mutations to eliminate RM recognition sites. (**b**) Expression of the *thermodcas9* gene. Lane 1: RT-PCR with *B. smithii* harboring pThermoCas9i_ NT (Mougiakos et al. 2017b) cDNA (positive control); lanes 2 and 3: RT-PCR of thermodcas9 cDNA with product size 124 bp from 2 independent transformants of *P. thermosuccinogenes* DSM 5809 harboring pThermoCas9i_ME; lane 4RT-PCR with *P. thermosuccinogenes* DSM 5809 wild type cDNA (negative control). (**c**) Expression of sgRNA. Lane 1: RT-PCR with *B. smithii* harboring pThermoCas9i_ NT (Mougiakos et al. 2017b) cDNA (positive control); lanes 2 and 3: RT-PCR of thermodcas9 cDNA with product size 169 bp from 2 independent transformants of *P. thermosuccinogenes* DSM 5809 harboring pThermoCas9i_ME. (**d**) Malic enzyme gene expression in *P. thermosuccinogenes* DSM 5809 harboring pThermoCas9i_ME was evaluated using qRT-PCR and expressed relative to the non-targeting control strain. Data represent the mean values of three biological replicates and the standard deviation. The level of significance of the differences was estimated by means of analysis of variance (ANOVA), with the statistically significant criterion being a *p*-value ≤ 0.05.

CRISPRi is a genetic perturbation tool that represses gene expression in bacteria. As a proof of concept, to study the efficacy of CRISPRi for gene suppression in *P. thermosuccinogenes*, we targeted the non-template strand of malic enzyme gene. We created pThermoCas9i vectors (using the pNW33N_RM5809 backbone) with ThermodCas9 under control of the *xylL* promoter from *B. smithii* and sgRNA under control of the intergenic 16S/23S rRNA promoter from *H. thermocellum* (Figure 4a), which was previously demonstrated to be functional for gene silencing in *H. thermocellum* [28]. The sgRNA was with a non-targeting spacer or a spacer targeting the promoter region of malic enzyme gene. The malic enzyme was chosen to validate if reducing its activity would increase the production of succinic acid. Targeting and non-targeting plasmids were isolated from *E. coli* HST04-S1-S2-S4 and electroporated to *P. thermosuccinogenes* DSM 5809. The colonies of *P. thermosuccinogenes* harboring non-targeting (Figure 4b,c) and targeting plasmid showed expression of the ThermodCas9 and sgRNA genes using RT-PCR. Next, qRT-PCR was performed to analyze the silencing efficacy of the transformants using 16S rRNA as housekeeping gene. The qRT-PCR analysis showed 75% reduction in the targeting gene expression, in comparison to the non-targeting transformants (Figure 4d). This indicates the suppression effectiveness of the targeting gene using CRISPRi with this specific sgRNA.

**Figure 5:**
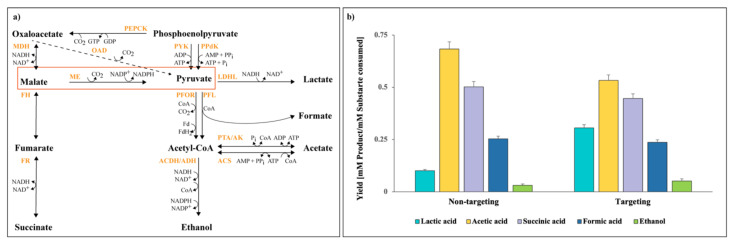
Impact of CRISPRi downregulation on the fermentation profile. (**a**) Illustration of *P. thermosuccinogenes* DSM 5809 metabolic pathways for conversion of phosphoenolpyruvate to major organic acids production, adapted from Koendjbiharie et al. [22]. ACDH—acetaldehyde dehydrogenase; ACS—acetyl-CoA synthase; ADH—alcohol dehydrogenase; AK—acetate kinase; FH—fumarate hydratase; FR—fumarate reductase; LDHL—lactate dehydrogenase; MDH—malate dehydrogenase; ME—malic enzyme; OAD—oxaloaceate decarboxylase; PEPCK—phosphoenolpyruvate carboxykinase; PFL—pyruvate-formate lyase; PFOR—pyruvate:ferredoxin oxidoreductase; PPdK—pyruvate kinase; PTA—phosphotransacetylase; PYK—pyruvate kinase. (**b**) Effects of CRISPRi-mediated repression on product formation of malic enzyme silencing transformants of both non-targeting and targeting plasmids. Data represent the mean values of three biological replicates and the standard deviation. The level of significance of the differences was estimated by means of analysis of variance (ANOVA), with the statistically significant criterion being a *p*-value ≤ 0.05.

Next, we cultured both the non-targeting and targeting transformants and performed HPLC analysis to obtain insight into their fermentation profiles. The major products of *P. thermosuccinogenes* were succinate, acetate, and formate, while lactate and ethanol were minor products (Figure 5a). It was evidently noted that repression of the gene encoding malic enzyme led to an effect on primarily lactate and acetate production in comparison to non-targeting cells (Figure 5b). At OD_600_ ~ 1.0, the acetate production decreased to 28% in contrast to lactate production, which increased by 33% in *P. thermosuccinogenes* with the malic enzyme gene repressed. The repression affected the pyruvate node directly, which may have driven the flux toward the closest lactate branch. Other products such as succinate, formate, and ethanol did not show any significant differences between the targeting and the non-targeting transformants (Figure 5b). In addition, no substantial changes were observed for malate and pyruvate production. In summary, qRT-PCR and HPLC analysis showed that reduction in malic enzyme gene expression results in a decrease in acetate and enhanced lactate production at both exponential and stationary growth phase of the fermentation. This shows that ThermodCas9, as a proof of concept, can silence metabolic genes in *P. thermosuccinogenes* with an impact on product formation. Further studies are needed to demonstrate the impact of different sgRNAs on repression of malic enzyme gene expression.

## 4. Discussion

*Pseudoclostridium thermosuccinogenes* DSM 5809 is an anaerobic thermophile with potential for succinate production [17,22]. Development of a thermophilic production process for succinic acid using renewable resources could be achieved via an engineered *P. thermosuccinogenes*. Therefore, we developed a reproducible electroporation protocol to introduce plasmid DNA in *P. thermosuccinogenes*. A series of pilot experiments were conducted with *P. thermosuccinogenes* DSM 5809, based on literature protocols for other clostridial species [6,43,44,45,46,47,48,49]. To study the genetic accessibility of *P. thermosuccinogenes*, *E. coli* DH5α-*Bacillus* shuttle vector pNW33N encoding chloramphenicol resistance marker [37] was chosen based on two vital features: i) the origin of replication to propagate in both *E. coli* (cloning host) and *Clostridium* (host of interest), and ii) limited availability of antibiotic markers (being a thermophile). Another *E. coli–Geobacillus* shuttle vector pUCG3.8 encoding the thermostable kanamycin nucleotidyl transferase gene [50] was also tested but failed to give transformants. The reasons might be that the plasmid with *Geobacillus* origin of replication was unable to propagate in clostridia or the presence of recognition sites 1, 2, and 3 (Table 1) in the plasmid, hampered DNA uptake. Furthermore, electroporation was performed using various growth states of cells (OD_600_ 0.4–1.2), electroporation buffers (SMP, glycerol, ddH_2_O), electroporation cuvettes (0.1 and 0.2 cm gap), and electrical parameters (field strength 2.5–15 kV/cm, time constant 5–20 ms). Unfortunately, none of the settings yielded transformants, suggesting the presence of RM systems correlated with improper DNA methylation or other critical factors such as the thick cell wall of Gram-positive bacteria, electroporation settings, and physiological state of the cells that hinder DNA uptake.

The plasmid pNW33N, previously introduced and isolated from *E. coli* DH5α (*dam^+^/dcm^+^*), was used to transform different intermediate *E. coli* strains (Acella, JM110, and HST04) prior their transformation in *P. thermosuccinogenes*. As a result, only pNW33N plasmid extracted from *E. coli* JM110 and HST04 yielded a few colonies with the electroporation protocol. Both strains have the *dam* and *dcm* genes deleted. The difference between the two strains is the additional deletion of genes encoding type I and IV RM systems in *E. coli* HST04 (Δ*mrr-hsdRMS-mcrBC*). The absence of type I RM genes is crucial to avoid different methylation pattern of plasmids to the clostridia methylation. Moreover, the presence of plasmids with a different methylation pattern from *P. thermosuccinogenes* could lead to the activation of native type IV RM systems in clostridia*,* which cleaves methylated DNA [11,15]. Therefore, the fact that only plasmids isolated from *dam*^−^ *dcm*^−^ strains such as *E. coli* HST04 and JM110 could be transformable in this thermophile, in contrast to other *E. coli* strains, hint at the presence of unidentified type IV RM systems in *P. thermosuccinogenes*.

To gain insight into the RM systems of *P. thermosuccinogenes*, PacBio methylation analysis was used to identify the presence of RM motifs in the genome. Three type I and one type III RM systems were annotated in the genome sequence data, and four methylated restriction sites were identified by the methylation analysis. Although methylcytosine modifications are known to occur in clostridia and to hamper transformation efficiencies [6,48], these were not observed in our analysis. It is known that methylcytosine detection is more difficult with PacBio sequence data (PMID: 23339471) and these may have been overlooked. The methylation analysis allowed us to search for the presence of putative RM recognition sites of *P. thermosuccinogenes* DSM 5809 in pNW33N plasmids. We identified three recognition sites that might potentially abolish the acquisition of pNW33N by *P. thermosuccinogenes*. Since few transformants were obtained when *E. coli* JM110 and HST04 were used as intermediate hosts, we hypothesized an escape strategy of the plasmids from the native RM systems enabling propagation into the host. This may also explain the low reproducibility and efficiency of the transformation protocol. To test this hypothesis, two strategies were followed. On one hand, the detected recognition sites were mutated, so they cannot further be recognized by the identified RM systems. On the other hand, the transforming plasmids were protected by in vivo methylation. In this approach, the native genes encoding type I RM systems from *P. thermosuccinogenes* DSM 5809 were mimicked in *E. coli* strains and plasmid DNA was isolated from these specific strains to overcome the restriction barrier. In the first strategy, the plasmid pNW33N_RM5809 (pNW33N lacking two of the three RM recognition sites; Figure 3a) was electroporated into *P. thermosuccinogenes* with improved reproducibility (i.e., reduced amount of non-successful electroporation experiments) compared with the pNW33N. However, the transformation efficiency (of the successful transformations) was not affected. In the second strategy, we observed improvements on reproducibility and on transformation efficiency. A transformation efficiency of 10^2^ CFU/µg DNA was achieved when the plasmid was in vivo methylated in an engineered *E. coli* HST04 strain, containing the three native methylation systems of *P. thermosuccinogenes*. This strategy has also significantly increased the transformation efficiency in other bacteria [12,17,19,43].

For *P. thermosuccinogenes* DSM 5809, SMRT PacBio sequencing revealed the presence of type I and type III restriction-modification systems in addition to four motifs with ^6m^A methylation. However, the sequencing data did not allow for linking which type I restriction system correlates to which specific motif. Given the easy access to MinION sequencing and initial reports on its application for methylation analysis, we attempted to perform a preliminary methylation analysis to see if we were able to detect DNA methylation patterns in the engineered *E. coli* HST04 strains. The improved transformation efficiencies of plasmid DNA isolated from HST04-S1-S2-S4 indicated that in vivo methylation of plasmid DNA seems to be active in *E. coli* HST04. We hypothesized that particularly the methylation of the recognition site ‘GATNNNNCTC’ of *P. thermosuccinogenes* in plasmid pNW33N is the reason of this observed improved efficiency. If such site is not methylated, it can hamper the genetic accessibility by being recognized by one of the RM systems that cleave foreign DNA. The preliminary methylation analysis of *E. coli* HST04-S1 strain with type I system has ^6m^A methylated in the sequence ‘GATNNNNCTC’ with significant p-value (Bonferroni < 1.05 × 10^-19^) compared to the wild type *E. coli* HST04 strain (Table S9, Supplementary Materials). Therefore, it was surprising to see that the effect of passaging plasmid DNA through this *E. coli* did not result in equally higher efficiencies as passaging through *E. coli* HST04-S1-S2-S4. It was equally surprising that the methylation analysis did not reveal any methylated motifs in HST04-S1-S2-S4 that showed improved transformation efficiencies. Apparently, the data handling pipeline used was not sensitive enough to detect the ^6m^A methylated ‘GATNNNNCTC’ motif that we expected to be present in *E. coli* HST04-S1-S2-S4. Hence, further optimization of data analysis is required, but the results with the HST04-S1 strain show that this may become a promising approach to identify the restriction systems accountable for genetic inaccessibility, although the underlying mechanism is not completely understood.

Once the transformation protocol was improved on transformation efficiency and reproducibility, we aimed at developing CRISPRi tool as a proof of concept. Our recent publication on the adaptation of CRISPRi on *H. thermocellum* using a thermostable Cas9 [28] opened new possibilities for transcriptional regulation of the thermophile *P. thermosuccinogenes*. Because of the close relatedness of the two clostridial species, we hypothesized that the CRISPRi plasmid used to downregulate metabolic genes from *H. thermocellum* might be functional in *P. thermosuccinogenes*. Dead Cas9-based CRISPRi allows the transcriptional regulation of the gene of interest without completely disrupting its function, resulting in less pleiotropic effects than gene knockouts [51,52]. We targeted the central metabolic gene encoding NADP^+^-dependent malic enzyme. With this, we successfully proved the functionality of the ThermoCas9i plasmid in *P. thermosuccinogenes*, achieving 75% reduction in the malic enzyme gene expression. The impact of variations of sgRNAs on gene silencing should be examined in future studies to better apply this genetic tool.

Finally, we showed that the silencing of the malic enzyme gene has an impact on the fermentation profile. It results in increased lactate production and declined acetate production. The malic enzyme is part of the malate shunt pathway together with malate dehydrogenase and phosphoenolpyruvate carboxykinase to produce pyruvate from phosphoenolpyruvate in *P. thermosuccinogenes*, as depicted in Figure 5a [53]. Because of the silencing, the pyruvate accumulation was lowered, which seems to direct the flux toward lactate production at the cost of acetate. We expected effects on malate or pyruvate concentrations, but no significant variations were observed. In addition, prominent changes in concentrations of other organic acids or ethanol were also not noticed. To improve the yields of desired products, such as succinate, knockout, or knockdown of the lactate dehydrogenase plus malate shunt pathway genes could be achieved. For instance, *H. thermocellum* YD02 strain was created with heterologous expression of pyruvate kinase and deletion of malic enzyme plus lactate dehydrogenase genes. This strain showed increase in ethanol and formate production [54]. Hence, we can apply dCas9 CRISPR system for *P. thermosuccinogenes* to manipulate other candidate genes and study its impact on succinate production with elimination of byproducts.

## 5. Conclusions

In summary, this work demonstrates the successful development of a genetic transformation procedure for *P. thermosuccinogenes*. We established a straightforward workflow to handle non-model organisms from genetic accessibility to developing silencing tool for transcriptional suppression of metabolic genes. The strategies made to overcome restriction barriers were the introduction of silent mutations and in vivo methylation of plasmid DNA. Moreover, we applied CRISPRi to downregulate the expression of the metabolic gene that encodes the malic enzyme affecting the metabolism of the organism. As a result, this is the first study of an effective genetic tool development and metabolic engineering for *P. thermosuccinogenes*, signifying advancement for this industrially relevant bacterium for production of green chemicals.

## Figures and Tables

**Table 1:** Methylome data of *P. thermosuccinogenes* DSM 5809. The percentage of these methylated recognition sites (motifs) in the genome denoted as % Modified. Motifs in the genome represent the number of times each motif appears in the genome. Methylated ^m6^A bases are in bold. Nucleotide code: B (C, G, or T), D (A, G, or T), N (any base), R (A or G), V (A, C, or G), and Y (C or T). *P. thermosuccinogenes* DSM 5809 genome sequence was assembled using data from Illumina HiSeq and Pacbio sequencing [21].

RM System	RM Type	Motifs	Modification Type	% Modified	Motifs in Genome
1	I	CACNNNNNNNTNGC/GCNANNNNNNNGTG	^m6^ A	95.09/88.47	876
2	I	GATNNNNCTC/GAGNNNNATC	^m6^ A	94.29/82.99	1630
3	I	TCABNNNNNNTARG/CYTANNNNNNVTGA	^m6^ A	90.24/85.79	697
4	III	CGAG	^m6^ A	66.04	10,352

## Data Availability

All data generated and analyzed in this study are included in this article and the Supplementary Materials.

## Supplementary Materials

The following are available online at https://www.mdpi.com/2076-2607/10/4/698/s1, Figure S1: Percent sporulation of *P. thermosuccinogenes* DSM5809 under phase contrast microscopy, Figure S2: Schematic representation of the cassettes of RM systems from *P. thermosuccinogenes* to be integrated in *E. coli* HST04, Figure S3: Colony PCR on single *P. thermosuccinogenes* colonies containing the pNW33N plasmid. Table S1: Bacterial strains used in the present study, Table S2: Plasmids used in the present study, Table S3: Spacers used in the present study, Table S4: Point mutations introduced in ThermoCas9i/pNW33n plasmid, Table S5: Codon usage of *P. thermosuccinogenes* DSM5809, Table S6: Primers used in the present study, Table S7: Cassette construction, Table S8: DNA methylation (*dam*/*dcm*) effects on electro-transformation efficiency when plasmids prepared from different *E. coli* hosts, Table S9: Minion sequencing data for motif GATNNNNCTC. Supplementary Section S1: Media preparation, Supplementary Section S2: Materials and Methods-Minion Sequencing. References [35,37,41,55,56,57] are cited in the Supplementary Materials.
