# Supplementary material for: Breaking the Restriction Barriers and Applying CRISPRi as a Gene Silencing Tool in Pseudoclostridium thermosuccinogenes
Source: Microorganisms. 2022 Mar 24;10(4):698. doi: 10.3390/microorganisms10040698 (PMC9044749; doi:10.3390/microorganisms10040698)
Supplement: Supplementary file 1 [file microorganisms-10-00698-s001.zip › microorganisms-1603118-supplementary.pdf]

microorganisms

MDPI

SUPPLEMENTARY DATA

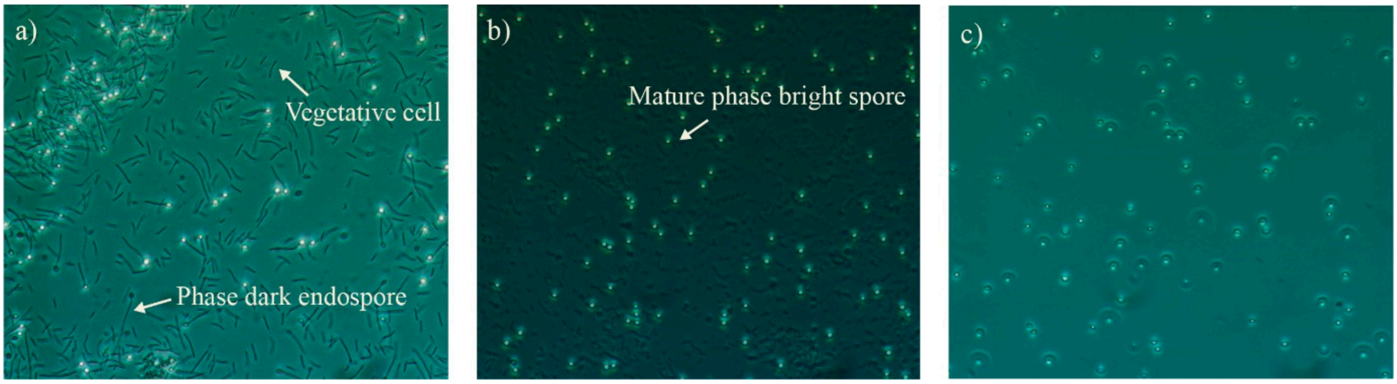

Figure S1. Percent sporulation of *P. thermosuccinogenes* DSM5809 under phase contrast microscopy **a)** Sporulation solution after 1 day in CP medium. **b)** Sporulation solution after 2 days in CP medium. **c)** Purified spores using 50% (*w*/*v*) solution of Histodenz, depicting > 95% purity.

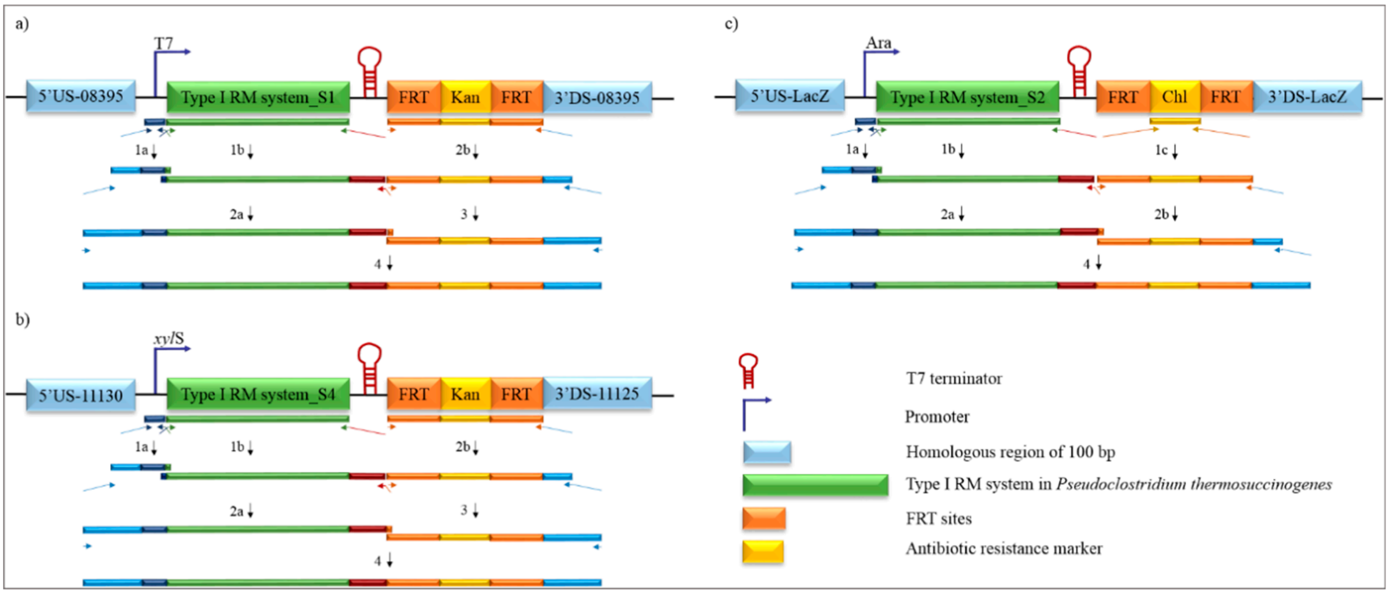

Figure S2. Schematic representation of the cassettes of RM systems from *P. thermosuccinogenes* to be integrated in *E. coli* HST04. **a)** The cassette is composed by the T7 promoter, the S1 type I RM system, the T7 terminator and the kanamycin resistance gene flanked by the *FRT* sites. The 100 bp long homology arms direct the cassette to the bifunctional isocitrate dehydrogenase kinase/phosphatase gene (08395). **b)** The cassette is composed of the XylS/Pm promoter, the S4 type I RM system, the T7 terminator and the kanamycin resistance gene flanked by the *FRT* sites. The upstream 100 bp region (5′US) targets the ArsR family transcriptional regulator (11130) and the downstream 100 bp homology region (3′DS) targets the arsenic transporter (11125). **c)** The cassette is composed of the arabinose promoter, the S2 type I RM system, the T7 terminator and the chloramphenicol resistance gene flanked by the *FRT* sites. The 100 bp long homology arms direct the cassette to the *lac*Z gene. Cassettes a and b were equally built. Three individual PCR reactions (1a, 1b, 2b) were performed using primers with the appropriate overhangs. Followed by an overlap-extension PCR (2a) and an extra single reaction (3). The products yielded by the last two reactions were combined in the last overlap-extension PCR (4) to obtain the complete cassette. Cassette c followed the same strategy as cassettes a and b with some slight differences. PCR reaction 1c is added and the reaction 3 is not necessary to be performed.

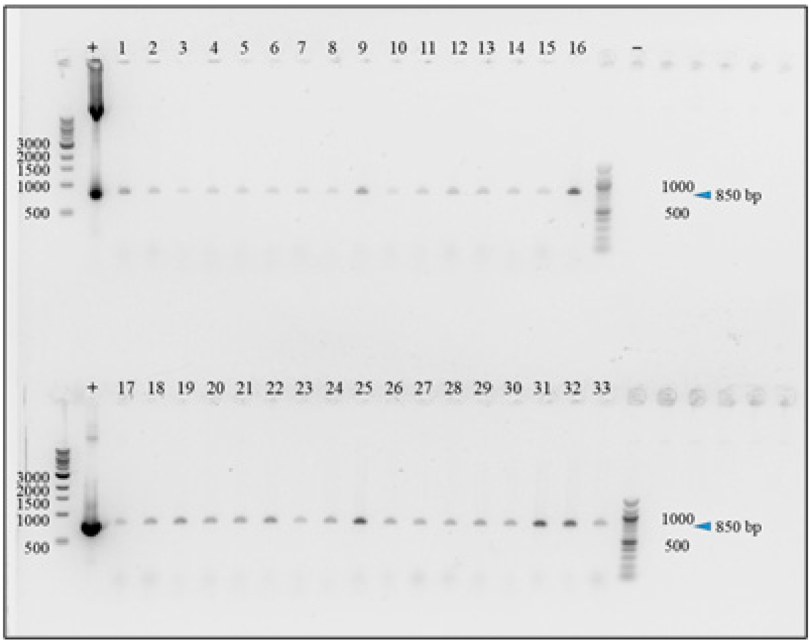

Figure S3. Colony PCR on single *C. thermosuccinogenes* colonies containing the pNW33N plasmid. Colonies were analyzed for the presence of 850 bp fragment, amplified from the chloramphenicol resistance gene using the primers BG10464 and BG10465. The positive control (+) is the pNW33N plasmid and the negative control (−) is the PCR mix without the DNA template.

Table S1. Bacterial strains used in the present study.

Strains	Description	Source
***Escherichia coli* DH5α**	*fhuA2 (argF-lacZ) U169 phoA glnV44 80 (lacZ)M15 gyrA96 recA1 relA1 endA1 thi^{-1} hsdR17 (Methylation status: hsdM+)*	New England Biolabs
***Escherichia coli* Acella**	*F^{-}ompT hsdSB (rB^{-} mB^{-}) gal dcm (DE3) ΔendA ΔrecA (Methylation status: hsdM−)*	Edge Bio
***Escherichia coli* JM110**	*psL (Strr) thr leu thi^{-1} lacY galK galT ara tonA tsx dam dcm supE44 Δ(lac-proAB) [F′ traD36 proAB lacIq ZΔM15] (Methylation status: hsdM+)*	[1]
***Escherichia coli* HST04**	*F^{-}, ara, Δ(lac-proAB) [Φ80d lacZΔM15], rpsL(str), thi, Δ(mrr-hsdRMS-mcrBC), ΔmcrA, dam, dcm (Methylation status: hsdM−)*	Clontech Laboratories (Takara)
***Escherichia coli* CGSC 7632**	*E. coli* CGSC 7632 with the pKD4-kan plasmid (*FRT*-kanamycin-*FRT* cassette)	[2]
***Escherichia coli* CGSC 7632**	*E. coli* CGSC 7632 with the pKD3-Chl plasmid (*FRT*-chloramphenicol-*FRT* cassette)	[2]
Pseudoclostridium thermosuccinogenes	DSM5809	DSMZ
***Escherichia coli* HST04-S1**	*E. coli* HST04 with the S1 type I RM system. The bifunctional isocitrate dehydrogenase kinase/phosphatase gene (08395) was disrupted	This study
***Escherichia coli* HST04-S2**	*E. coli* HST04 with the S2 type I RM system. The *lacZ* gene was disrupted	This study
***Escherichia coli* HST04-S4**	*E. coli* HST04 with the S4 type I RM system. Transcriptional regulator gene *arsR* (11130) and the arsenic transporter gene (11125) were disrupted	This study
***Escherichia coli* HST04-S1-S2-S4**	*E. coli* HST04 with the S1, S2 and S4 type I RM systems	This study
***Escherichia coli* HST04-S1-S2-S4**	*E. coli* HST04 with pThermoCas9i-NT	This study
***Escherichia coli* HST04-S1-S2-S4**	*E. coli* HST04 with pThermoCas9i-ME	This study
**DSM 5809 (pNW33n)**	*P. thermosuccinogenes* with pNW33n	This study
**DSM 5809 (pNW33n_RM5809)**	*P. thermosuccinogenes* with pNW33n_RM5809	This study
**DSM 5809 (pThermoCas9i_NT)**	*P. thermosuccinogenes* with pThermoCas9i-NT	This study
**DSM 5809 (pThermoCas9i_ME)**	*P. thermosuccinogenes* with pThermoCas9i-ME	This study

Table S2. Plasmids used in the present study.

Plasmid	Primers	Description
**pNW33n**	-	*E. coli-Bacillus* shuttle vector pNW33n containing origin of replication functional in *H. thermocellum* and chloramphenicol/thiamphenicol antibiotic resistance [3]
**pNW33n_RM5809**	BG12510 and BG12511	Plasmid without the RM recognition sites of *P. thermosuccinogenes* DSM5809
	BG12512 and BG12513	
	BG12514 and BG12515	
	BG12516 and BG12517	
**pThermoCas9i_NT**	BG12508 and BG12518	Plasmid with the ThermodCas9 and the non-targeting sgRNA, which is under the 16S/23S intergenic promoter of *H. thermocellum* DSM1313
	BG12510 and BG12511	
	BG12512 and BG12513	
	BG12514 and BG12515	
	BG12516 and BG12517	
	BG12519 and BG12526	
	BG12527 and BG12509	
**pThermoCas9i_ME**	BG12508 and BG12518	Plasmid with the ThermodCas9 and the targeting sgRNA under the 16S/23S intergenic promoter of *H. thermocellum* DSM1313. The sgRNA with spacer targeting promoter of malic enzyme gene
	BG12509 and BG12523	
	BG12510 and BG12511	
	BG12512 and BG12513	
	BG12514 and BG12515	
	BG12516 and BG12517	
	BG12519 and BG12520	

Table S3. Spacers used in the present study.

Description	Spacer Sequences
**Non-targeting**	5′GCTAGTCTCAAGGTCATCAGTAGCT3′
**Spacer targeting the promoter of the NAD dependent malic enzyme**	5′ATTAAGATTTCCTATATAGATCTTA 3′

Table S4. Point mutations introduced in ThermoCas9i / pNW33n plasmid.

Plasmid Name	Plasmid Feature	Recognition Site in the Forward Strand (5′→ 3′)	Location Inside Thermodcas9 Plasmid (bp)	Mutation	Location of Mutation Inside the Protein (bp)	Amino Acid
**pNW33N and pThermoCas9i**	*repB* gene	GATNNNNNCTC	2124–2133	GCT→GCC	568	Alanine
**pNW33N and pThermoCas9i**	pNW33N backbone	GATNNNNNCTC	3124–3133	**A →C**	-	-
**pThermoCas9i**	ThermodCas9 gene	CYTANNNNNNVTGA	5079–5092	CC**T**→CC**A**	927	Proline
		GATNNNNNCTC	6016–6025	GG**C**→GG**T**	1860	Glycine
		GATNNNNNCTC	6359–6368	CC**G**→CC**A**	2196	Proline
**pNW33N and pThermoCas9i**	Promoter of the *cat* gene	GATNNNNNCTC	1454–1463	**-**	-	-

Table S5. Codon usage of *P. thermosuccinogenes* DSM5809.

Amino Acid	Codon	Fraction	Amino Acid	Codon	Fraction	Amino Acid	Codon	Fraction
**Ala**	GCG	0.12	**Pro**	CCG	0.22	**Gly**	GGG	0.18
	GCA	0.33		CCA	0.29		GGA	0.33
	GCT	0.32		CCT	0.30		GGT	0.25
	GCC	0.24		CCC	0.19		GGC	0.25
**Thr**	ACG	0.13	**Gln**	CAG	0.46	**His**	CAT	0.68
	ACA	0.33		CAA	0.54		CAC	0.32
	ACT	0.25	**Arg**	AGG	0.23	**Ile**	ATA	0.38
	ACC	0.29		AGA	0.23		ATT	0.35
**Glu**	GAG	0.34		CGG	0.19		ATC	0.27
	GAA	0.66		CGA	0.12	**Lys**	AAG	0.37
**Phe**	TTT	0.62		CGT	0.11		AAA	0.63
	TTC	0.38		CGC	0.12	**Met**	ATG	1.00
**Tyr**	TAT	0.70	**Cys**	TGT	0.46	**Asn**	AAT	0.63
	TAC	0.30		TGC	0.54		AAC	0.37

**Leu**	TTG	0.19	**Asp**	GAT	0.67	**Ser**	AGT	0.11
	TTA	0.20		GAC	0.33		AGC	0.18
	CTG	0.17	**Val**	GTG	0.19		TCG	0.09
	CTA	0.09		GTA	0.28		TCA	0.22
	CTT	0.23		GTT	0.32		TCT	0.18
	CTC	0.13		GTC	0.22		TCC	0.22
**Trp**	TGG	1.00						
**Stop**	TGA	0.39						
	TAG	0.17						
	TAA	0.44						

Table S6. Primers used in the present study.

Primers used for plasmid and cassette construction.

Primer Code	Sequence (5′-3′)
**BG10829**	TCATGACCAAAATCCCTTAACGTG
**BG10830**	CACGTTAAGGGATTTTGGTCATGA
**BG11739**	CCATTTTTGTTTAATCCTGTCGACGCCTATGTCGGCTTTGAG
**BG11740**	CCAGCTGGATACTAGCGTCATGGGATCCCCTCAGGTCCTTTCCTTTG
**BG11746**	AGGCGTCGACAGGATTAAACAAAAATGG
**BG11747**	GGATCCCATGACGCTAGTATCCAGCTGGGTCATAGTTCCCCTGAGATTATCG
**BG12493**	AATCTATAGATGAAATCATTTGTATATCTCCTTCTTAAAGTTAAA
**BG12494**	TTAACTTTAAGAAGGAGATATACAAATGATTTCATCTATAGATTATTTTCTC
**BG12495**	CAAAAAACCCCTCAAGACCCGTTTAGAGGCCCCAAGGGGTTATGCTATTACACAACCTCTTCCAATG
**BG12496**	TAGCATAACCCCTTGGGGCCTCTAAACGGGTCTTGAGGGGTTTTTTG GTGTAGGCTGGAGCTGCTTC
**BG12502**	AACTTTATCTTGAAAGTTAATCATTATGTTCATGACTCCATTATTATTG
**BG12503**	ATAATGGAGTCATGAACATAATGATTAACTTTCAAGATAAAGTTAA
**BG12504**	CAAAAAACCCCTCAAGACCCGTTTAGAGGCCCCAAGGGGTTATGCTA TTATATCACCTCGACTTTGTC
**BG12508**	ACAACGCCAAGCGATATCG
**BG12509**	TTGTCAGATATGGCCCAAGGATTTC
**BG12510**	GAAATCCTTGGGCCATATCTGACAA
**BG12511**	CTTTGAGTGAGCTGCTACCG
**BG12512**	CGGTAGCAGCTCACTCAAAG
**BG12513**	GGTGTCGTCTGGCAAGTTAAG
**BG12514**	CTTAACTTGCCAGACGACACC
**BG12515**	ACGTTCTGAACCTAATCCTAAATATTCA
**BG12516**	TGAATATTTAGGATTAGGTTCAGAACGT
**BG12517**	CGATATCGCTTGGCGTTGT
**BG12518**	TTCTCAAAGCCGACATAGGCGTCGACAGGATTAAACAAAAATGG
**BG12519**	TTTTGTTTAATCCTGTCGACGCCTATGTCGGCTTTGAG
**BG12520**	TAAGATCTATATAGGAAATCTTAATCCTCAGGTCCTTTCCTTTG
**BG12523**	ATTAAGATTTCCTATATAGATCTTAGTCATAGTTCCCCTGAGATTATCG
**BG12526**	CCAGCTGGATACTAGCGTCATGGGATCCCCTCAGGTCCTTTCCTTTG
**BG12527**	GGATCCCATGACGCTAGTATCCAGCTGGGTCATAGTTCCCCTGAGATTATCG
**BG12556**	ATTCGCGATGCTGATTGGAAAATTCGCGGGATTCCTGCGGACTTAGAAGATAATACGACTCACTATAGGGGAAT
**BG12557**	TAGCCTGGCAGGGTCAGGAAATCAATTAACTCATCGGAAGTGGTGATCTGCATATGAATATCCTCCTTAGTTCCTATTCC
**BG12558**	CTTTAAGAAGGAGATATACAATGTCTAAATTAACATTGCAAG
**BG12559**	CAAAAAACCCCTCAAGACCCGTTTAGAGGCCCCAAGGGGTTATGCTA TTATATATTTACTCTAATACTTCCTGT
**BG12560**	GTGTAGGCTGGAGCTGCTTCGAAGTTCCTATACTTTCTAGAGAATAGGAACTTCGGAATAGGAACTTCGGGTCCCCAATAATTACGATTTAC
**BG12561**	CATATGAATATCCTCCTTAGTTCCTATTCCGAAGTTCCTATTCTCTAGAAAGTATAGGAACTTCGACAATTGTCTCAGGTCGAGGT
**BG12562**	GGTGGCCGTGGTGTCGGCTCCGCCGCCTTCATACTGCACCGGGCGGGAAGCATATGAATATCCTCCTTAGTTCCTATTCC
**BG12567**	ACGGGTTGTTACTCGCTC
**BG12568**	TAGCCTGGCAGGGTCAGGAAATCAATTAACTCATCGGAAGTGGTGATCTGCATATGAATATCCTCCTTAGTTCCTATTCC
**BG12572**	TGTCATTTCTGTTACCCATCCAATTGTTCAAAATTCTTGCTGATGAAACCCAGAAGGAAACCCTCATTCG
**BG12573**	CGCTTCTGCGGGAGATTTCAGCAGCGCCATATCGTAGTTCTGCGGAATATCATATGAATATCCTCCTTAGTTCCTATTCC

Primers used to verify the presence of the pNW33N.

Primer Code	Sequence (5′-3′)
**BG10464**	AACTCTCCGTCGCTATTGTAACCA
**BG10465**	TATGCGTGCAACGGAAGTGAC

Primers used to sequence pThermodCas9i plasmids.

Primer Code	Sequence (5′-3′)	Characteristics
**BG11015**	GATGAATTAGCCCGCATCC	
**BG11016**	CACCCGTTTTAAAGGTCTTTTAT	
**BG11017**	ACCGAAAGAAAAACGAAACTG	
**BG11018**	CGAGTCACCGCCTTCTATC	
**BG11019**	GATGAAAGGGATCTTGCCAAAC	Particularly used to sequence the spacer
**BG11021**	GCTGTAATAATGGGTAGAAGGTA	
**BG11022**	CCACCATAGGAGATTAACCTTT	
**BG11023**	CTTTTTGTCTTGTCCACTAAAAC	
**BG11024**	TGGCGTTTTTCCATAGGC	
**BG11025**	CACGTTAAGGGATTTTGGTCATGA	

Primers used in the RNA reverse transcriptase PCR.

Primer Code	Sequence (5′-3′)
**BG11636**	ATCGGCATTACGTCTATCGG
**BG11637**	AACACGAAGCTGCCAGAC
**BG11642**	CAGCTGGGTCATAGTTCC
**BG11643**	CCTAAGAGTGGGGAATGC

Primers used in the Quantitative Real Time PCR.

Primer Code	Sequence (5′-3′)
**BG10427**	GCAATGGAGGAAACTCTGAC
**BG10428**	ACCCAGTAATTCCGGACAAC
**BG18976**	GGAAGGGCAAAATTGAAGTTAT
**BG18977**	CGAGGTTCCATCTTCTTGTC

Table S7. Cassette construction.

Cassette	Primers	Template	Fragment	Overlap Primers	Overlap PCR Templates	Fragment
**US0835_T7_S1_FRT_kan_FRT_DS0835**	BG12567 and BG12493	BG11816 (GenArt)	T7 promoter	BG12569 and BG12499	T7 promoter and S1 gene cluster	US0835_T7 _S1
	BG12494 and BG12495	gDNA *P. thermosuccinogenes* DSM 5809	S1 gene cluster			
	BG12496 and BG12568	pKD4 plasmid	*FRT-kan-FRT*	BG12570 and BG12571	US0835_T7 _S1 and *FRT-kan-FRT*	**US0835_T7_S1_FRT_kan_FRT_DS0835**
**USlacZ_araB_S2_FRT_Chl_FRT_DSlacZ**	BG12556 and BG12557	BG11818 (GenArt)	*araB* promoter	BG12563 and BG12499	araB promoter and S2 gene cluster	USlacZ_araB_S2
	BG12558 and BG12559	gDNA *P. thermosuccinogenes* DSM 5809	S2 gene cluster			
	BG12496 and BG12562	pKD3 plasmid	FRT-chl-FRT	BG12565 and BG12566	USLacZ_AraC_pm_S2 and FRT-chl-FRT	**USlacZ_araB_S2_FRT_chl_FRT_DSlacZ**
**US11130_Pm_S4_FRT_kan_FRT_DS11125**	BG12572 and BG12502	BG11820 (GenArt)	Pm promoter	BG12574 and BG12499	Pm promoter and S4 gene cluster	US11130_Pm_S4
	BG12503 and BG12504	gDNA *P. thermosuccinogenes* DSM 5809	S4 gene cluster			
	BG12496 and BG12573	pKD4 plasmid	*FRT-kan-FRT*	BG12575 and BG12576	US11130_Pm_S4 and *FRT-kan-FRT*	**US11130_Pm_S4_FRT_kan_FRT_DS11125**

Table S8. DNA methylation (*dam*/*dcm*) effects on electro-transformation efficiency when plasmids prepared from different *E. coli* hosts.

E. coli host	DNA methylation	Average CFU/ µg of plasmid pNW33n	Average CFU/ µg of plasmid pNW33n_RM5809
**DH5α**	*dcm⁺ /dam⁺*	-	-
**Acella**	*dcm⁻ /dam⁺*	-	-
**JM110**	*dcm⁻ /dam⁻*	3	2
**HST04**	*dcm⁻/dam⁻/hsdRMS*	5	2

Table S9. Minion sequencing data for motif GATNNNNCTC.

Strains	bonferroni-corrected *p-value**				
	E. coli HST04_S1	*E. coli* HST04_S2	*E. coli* HST04_S4	*E. coli* HST04_S1+S2+S4	*E. coli* HST04
***E. coli* HST04_S1**	1				
***E. coli* HST04 _S2**	1.10E-20	1			
***E. coli* HST04 _S4**	7.88E-20	1	1		
***E. coli* HST04_S1+S2+S4**	1.96E-20	1	1	1	
***E. coli* HST04 (control)**	1.05E-19	1	1	1	1

**p-value* < 0.05 indicates significant value.

Section S1. Media Preparation

CP Broth Medium

The stock solutions, the trace element solution (1000×) and the vitamin solution (1000×) were prepared in dH_2O water, following the composition depicted in **Table S10.1**, **S10.2** and **S10.3**, respectively.

Table S10.1. Formulation of stock solutions of CP broth medium.

Stock Solution	Reagent	Amount to Add (*w*/*v* %)	Comment
**1**	KH_2PO_4	2.72	-
**2**	$Na_2\ HPO_4{\cdot}2H_2O$	3.56	-
**3**	NH_4Cl	2.4	-
	NaCl	2.4	
	$MgCl_2{\cdot}6H_2O$	0.8	
**4**	$CaCl_2{\cdot}2H_2O$	1.1	-
**5**	$NaHCO_3$	8	-
**6**	$Na_2S{\cdot}9H_2O$	24.02	Anaerobic, flushed and pressurized bottle with N_2. Store in the dark. Make fresh stock every month since Na_2S is not very stable in water.
**7**	Resazurin	0.05	-

Table S10.2. Trace elements composition (1000×).

	Trace Element	Amount to Add (mg/L)
**Acid stock solution (I)**	HCl (50 mM)	1800
	H_3BO_3 (1 mM)	61.8
	$MnCl_2$ (0.5 mM)	61.25
	$FeCl_2$ (7.5 mM)	943.5
	$CoCl_2$ (0.5 mM)	64.5
	$NiCl_2$ (0.1 mM)	12.86
	$ZnCl_2$ (0.5 mM)	67.7
	$CuCl_2$ (0.1 mM)	13.35
**Alkaline stock solution (II)**	NaOH (10 mM)	400
	Na_2SeO_3	17.3
	Na_2WO_4	29.4
	Na_2MoO_4	20.5

Table S10.3. Vitamins solution (1000×).

Vitamin	Amount to Add (mg/L)
**Biotin**	20
**Nicotinamid**	200
**p-Aminobenzoic acid**	100
**Thiamin (Vitamin B1)**	200
**Panthotenic acid**	100
**Pyridoxamine**	500
**Cyanocobalamine (Vitamin B12)**	100
**Riboflavine**	100

The calculated amounts of each solution were added to dH_2O, according to the percentages presented in **Table S10.4**. Subsequently, 0.5 (*w*/*v*) % of yeast extract was added and dissolved thoroughly until the solution was completely homogenous. The pH was set at 7–7.2 at room temperature and. The final volume was adjusted with dH_2O. The media was boiled to remove oxygen up to 20 s. Immediately after, the media was placed on ice-water and cool to room temperature under an oxygen-free N_2 flow for 30 min, preventing oxygen diffusion into the medium. The CP medium was dispensed into serum vials, which in turn were flushed and pressurized with sterile 80% N_2 – 20% CO_2 to 1.5–1.8 pressure. The media was sterilized by autoclaving for 20 min at 15 psi (1.05 kg/cm^2).

Table S10.4. Formulation of CP broth medium.

Stock Solution	Amount to Add (*v*/*v* %)
**7**	0.1
**1**	1.5
**2**	1.5
**3**	1.25
**Acid stock solution (I)**	0.1
**Alkaline stock solution (II)**	0.1

Before inoculation, the vials were supplemented with the stock solutions presented in **Table S10.5**: 1% (*v*/*v*) of the calcium and vitamin solution, 2% (*v*/*v*) of glucose solution and 5% (*v*/*v*) of the reducing solution.

Table S10.5. Sterile anaerobic stock solutions.

Stock Solution	Compound	Amount to Add	Comment
**Carbon source**	Glucose	250 g/L	Anaerobic, flushed and pressurized bottle with N_2
**Calcium / Vitamin solution**	Stock solution 4	45 mL	Anaerobic, flushed and pressurized bottle with 80% N_2 – 20% CO_2.
	Vitamin solution	4.5 mL	Vitamin solution is added after autoclaving with sterile syringe filter of 0.2μm pore size.
**Reducing solution**	Stock solution 5	50 mL	Anaerobic, flushed and pressurized bottle with 80% N_2 – 20% CO_2. Solution 6 is added after autoclaving.
	Stock solution 6	1 mL	
	L-cysteine hydrochloride	0.5 g/L	

CTFUD Medium

The calculated amounts of each solution were added to dH_2O, according to the percentages presented in **Table S10.6** and dissolved thoroughly until the solution was completely homogenous. The pH was set at 7–7.2 at room temperature and. The final volume was adjusted with dH_2O. The media was boiled to remove oxygen up to 20 s. Immediately after, the media was placed on ice-water and cool to room temperature under an oxygen-free N_2 flow for 30 min, preventing oxygen diffusion into the medium. The CP medium was dispensed into serum vials, which in turn were flushed and pressurized with sterile 80% N_2 – 20% CO_2 to 1.5–1.8 pressure. The media was sterilized by autoclaving for 20 min at 15 psi (1.05 kg/ cm^2). After autoclavation, glucose was added at 2% (*v*/*v*), and any antibiotic was added if necessary.

Table S10.6. Formulation of CTFUD medium.

Reagent	Amount to Add (% *w/v*)	Comment
**$(NH_4)_2SO4$**	0.13	
**KH_2PO_4**	0.15	Stock solution 1 of CP medium can be used
**$CaCl_2{\cdot}2H_2O$**	0.013	Stock solution 4 of CP medium can be used
**Cysteine HCl**	0.05	
**MOPS sodium salt**	1.156	
**$FeSO_4{\cdot}7H_2O$**	0.0001	
**$MgCl_2{\cdot}6H_2O$**	0.26	
**Yeast extract**	0.45	
**Na-resazurin solution (0.1% *w/v*)**	0.5 Ml	Stock solution 7 of CP medium can be used
**$C_6H_5Na_3O_7{\cdot}2H_2O$**	0.3	

Sporulation Media

26.31 g of Modified AEA Sporulation Medium Base (catalogue number 17170, Sigma Aldrich) was dissolved in an Erlenmeyer flask with 1L of dH_2O. The media was boiled to remove oxygen up to 20 s. Immediately after, the media was placed on ice-water and cool to room temperature under an oxygen-free N_2 flow for 30 min, preventing oxygen diffusion into the medium. The CP medium was dispensed into serum vials, which in turn were flushed and pressurized with sterile 80% N_2 – 20% CO_2 to 1.5–1.8 pressure. The media was sterilized by autoclaving for 20 min at 15 psi (1.05 kg/ cm^2). After autoclavation, the vials were supplemented with the stock solutions presented in **Table S10.7**. 4% (*v/v*) of glucose solution, 1.33% (*v/v*) of sodium carbonate (Na_2CO_3), 1.33% (*v/v*) dropwise of cobalt chloride hexahydrate ($CoCl_2{\cdot}6H_2O$). Later, 1.33% (*v/v*) of the freshly prepared sodicum ascorbate solution was added to the media. The media was incubated for 10 min at 55 °C prior 1 mL of the culture was added.

Table S10.7. Formulation of stock solutions for sporulation medium.

Reagent	Amount to Add (% *w/v*)	Comment
**Glucose**	10	Anaerobic, flushed and pressurized bottle with N2 and autoclave.
**Na_2CO_3**	7	Filter sterilized solution using syringe filter of 0.2 μm pore size.
**$CoCl_2{\cdot}6H_2O$**	0.58	Filter sterilized solution using syringe filter of 0.2 μm pore size.
**$C_6H_7O_6Na$**	1.5	Filter sterilized solution using syringe filter of 0.2 μm pore size. It must be freshly prepared.

Section S2. Materials and Methods – Minion Sequencing

Sample Preparation

The generated *E. coli* HST04 strains with the integrated cassettes (S1, S2, S4 or S1-S2-S4) were used to correlate individual integrated type I RM systems in *E. coli* HST04 to each identified motif using Minion sequencing. These *E. coli* strains were grown at 37 °C to an OD_{600} ~ 0.4–0.5 and induced for proper expression of the methylation genes. Type I RM system_S1 was induced with 0.1 mM IPTG, type I RM system_S2 was induced with 0.1% arabinose and type I RM system_S4 was induced with 1 mM m-tuloic acid. After induction, genomic DNA isolation was done using the MasterPure™ Gram Positive DNA Purification Kit (Lucigen). The purified DNA of each strain was further used for Minion sequencing using the 1D Native barcoding genomic DNA Kit (with EXP-NBD104, EXP-NBD114 and SQK-LSK109). The data generated from this experiment was further processed and analyzed as explained in the next sections.

Data Processing

First, we used Deepbinner for demultiplexing barcodes to the raw data (fas5) produced by Oxford Nanopore [4]. Then, base calling was performed using Guppy v2.1.3 with the "flip-flop" algorithm and quality control was performed with MinIONQC (qscore > 7) [5]. Trimming of barcode-adapter sequences, introduced during library preparation, was performed using Porechop v0.2.4 (https://github.com/rrwick/Porechop (accessed on 1 January 2022)). Using Tombo [6] we filtered reads without read identifier

(resulting from the demultiplexing procedure) using -- *preprocess annotate_raw_with_fastqs*. Secondly, we continued with the resquiggling -- *resquiggle* where it associates the raw signals and base call to the reference genome *E.coli* HST04. Lastly, we detected modifications using the model for DNA *N*6-methyldeoxyadenosine (6mA), where we retrieved a report from the position with the highest modification fraction reported by Tombo. Then, the rest were further divided into four groups according to the nucleotide base with the highest modification fraction in wig format.

We obtained the list of motifs combination for unspecific sequences and then used blastn on the *E. coli* genome reference to obtain the chromosomal positions where the motifs with 6mA modification were positioned. For this, we used in-house python and R scripts (https://github.com/genid/nanopore-analysis-Pseudoclostridium (accessed on 1 January 2022)).

Data Analysis

For the data analysis we used the matched 6mA frac levels for each of the motifs. For each of the comparison we performed a non-parametric test with Wilcoxon also called Mann–Whitney U test (similar to a *t*-test used to compare mean distributions).

References

1. Yanisch-Perron, C., Vieira, J., Messing, J. Improved M13 phage cloning vectors and host strains: Nucleotide sequences of the M13mp18 and pUC19 vectors. *Gene,* **1985**, *33*, 103–119.
2. Olson, D.G., Lynd, L.R. Transformation of *Clostridium thermocellum* by electroporation. *Meth. Enzymol.* **2012**, *510*, 317–330.
3. Datsenko, K.A., Wanner, B.L. One-step inactivation of chromosomal genes in *Escherichia coli* K-12 using PCR products. *Proc. Natl. Acad. Sci. U.S.A,* **2000**, *97*, 6640–6645.
4. Wick, R.R., Judd, L.M., Holt, K.E. Deepbinner: Demultiplexing barcoded Oxford Nanopore reads with deep convolutional neural networks. *PLoS Comput. Biol.,* **2018**, *14*, e1006583.
5. Lanfear, R., Schalamun, M., Kainer, D., Wang, W., Schwessinger, B. MinIONQC: Fast and simple quality control for MinION sequencing data. *Bioinformatics* **2019**, *35*, 523–525.
6. Stoiber, M., Egan, R., Lee, J.E., Celniker, S., Neely, R.K., Loman, N., Pennacchio, L.A., Brown, J. De novo Identification of DNA Modifications Enabled by Genome-Guided Nanopore Signal Processing. *BioRxiv* **2017**.
